# Supplementary material for: Critical enzymes for biosynthesis of cucurbitacin derivatives in watermelon and their biological significance
Source: Commun Biol. 2020 Aug 14;3:444. doi: 10.1038/s42003-020-01170-2 (PMC7429850; doi:10.1038/s42003-020-01170-2)
Supplement: Supplementary file 3 — Description of Additional Supplementary Files [file 42003_2020_1170_MOESM3_ESM.pdf]

Description of Additional Supplementary Files

File Name: **Supplementary Data 1**
Description Dose dependent neuronal results.

File Name: **Supplementary Data 2**
Description HPLC results of wounding and *ACTs* over expression.
