# Supplementary material for: Critical enzymes for biosynthesis of cucurbitacin derivatives in watermelon and their biological significance
Source: Commun Biol. 2020 Aug 14;3:444. doi: 10.1038/s42003-020-01170-2 (PMC7429850; doi:10.1038/s42003-020-01170-2)
Supplement: Supplementary file 4 — Supplementary Information [file 42003_2020_1170_MOESM4_ESM.pdf]

Supplementary information

Supplementary Figures

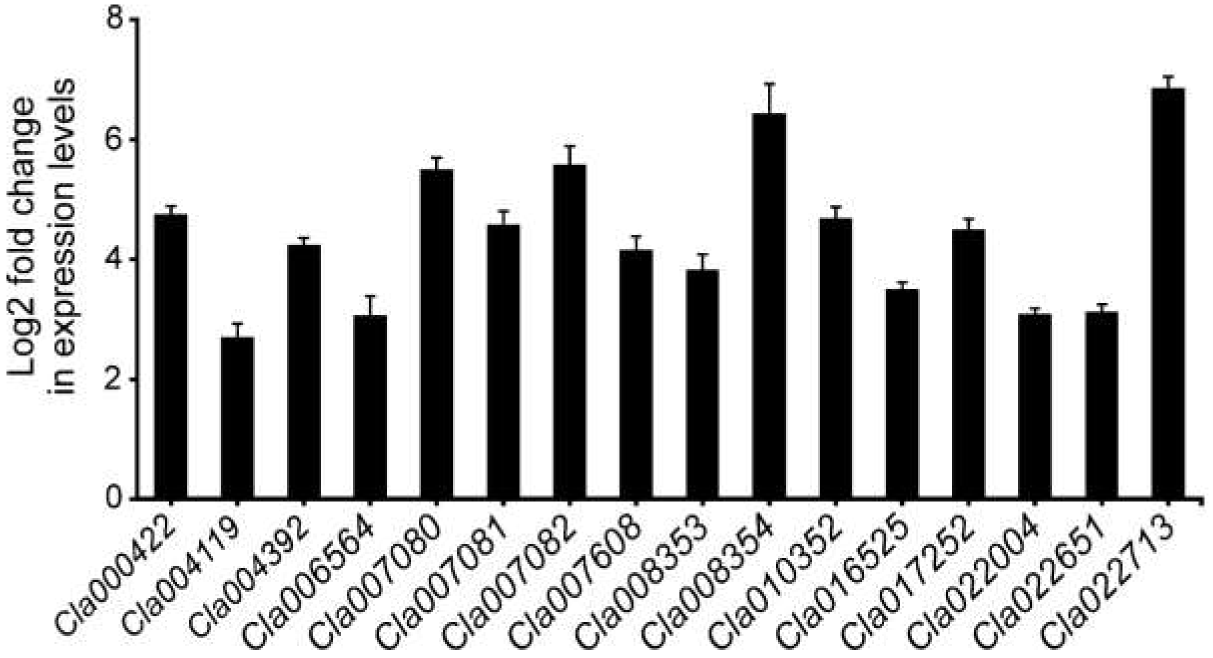

Supplementary Figure 1. RT-qPCR validation of up-regulated in PI532627 compare to PI536451. RT-qPCR analysis results showing log2 fold change in expression for each gene expected to involve in cucurbitacins biosynthetic pathway.

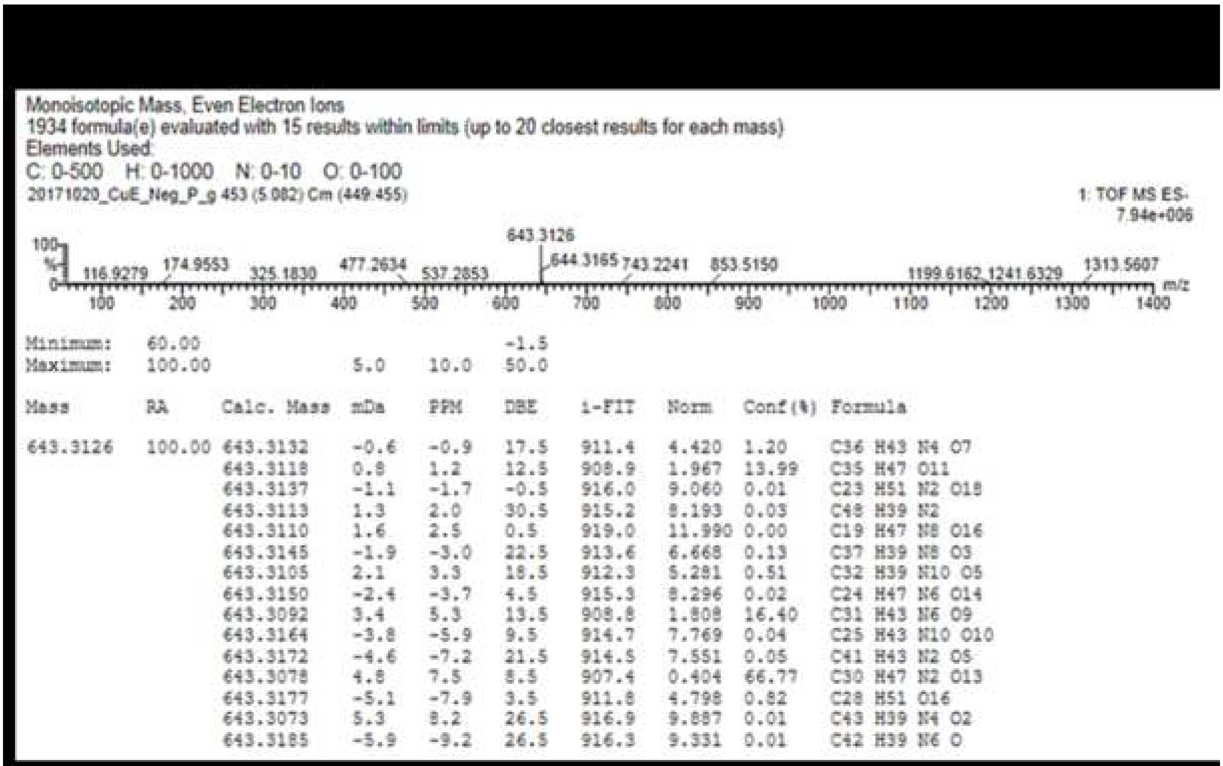

Monoisotopic Mass, Even Electron Ions
1934 formula(e) evaluated with 15 results within limits (up to 20 closest results for each mass)
Elements Used:
C: 0-500 H: 0-1000 N: 0-10 O: 0-100
20171020_CuE_Neg_P_g 453 (5.082) Cm (449:455)

1: TOF MS ES-
7.94e+006

Mass	RA	Calc. Mass	mDa	PPM	DBE	i-FIT	Norm	Conf(%)	Formula
Minimum:	60.00				-1.5				
Maximum:	100.00		5.0	10.0	50.0				
643.3126	100.00	643.3132	-0.6	-0.9	17.5	911.4	4.420	1.20	C36 H43 N4 O7
		643.3118	0.8	1.2	12.5	908.9	1.967	13.99	C35 H47 O11
		643.3137	-1.1	-1.7	-0.5	916.0	9.060	0.01	C23 H51 N2 O18
		643.3113	1.3	2.0	30.5	915.2	8.193	0.03	C48 H39 N2
		643.3110	1.6	2.5	0.5	919.0	11.990	0.00	C19 H47 N8 O16
		643.3145	-1.9	-3.0	22.5	913.6	6.668	0.13	C37 H39 N8 O3
		643.3105	2.1	3.3	18.5	912.3	5.281	0.51	C32 H39 N10 O5
		643.3150	-2.4	-3.7	4.5	915.3	8.296	0.02	C24 H47 N6 O14
		643.3092	3.4	5.3	13.5	908.8	1.808	16.40	C31 H43 N6 O9
		643.3164	-3.8	-5.9	9.5	914.7	7.769	0.04	C25 H43 N10 O10
		643.3172	-4.6	-7.2	21.5	914.5	7.551	0.05	C41 H43 N2 O5
		643.3078	4.8	7.5	8.5	907.4	0.404	66.77	C30 H47 N2 O13
		643.3177	-5.1	-7.9	3.5	911.8	4.798	0.82	C28 H51 O16
		643.3073	5.3	8.2	26.5	916.9	9.887	0.01	C43 H39 N4 O2
		643.3185	-5.9	-9.2	26.5	916.3	9.331	0.01	C42 H39 N6 O

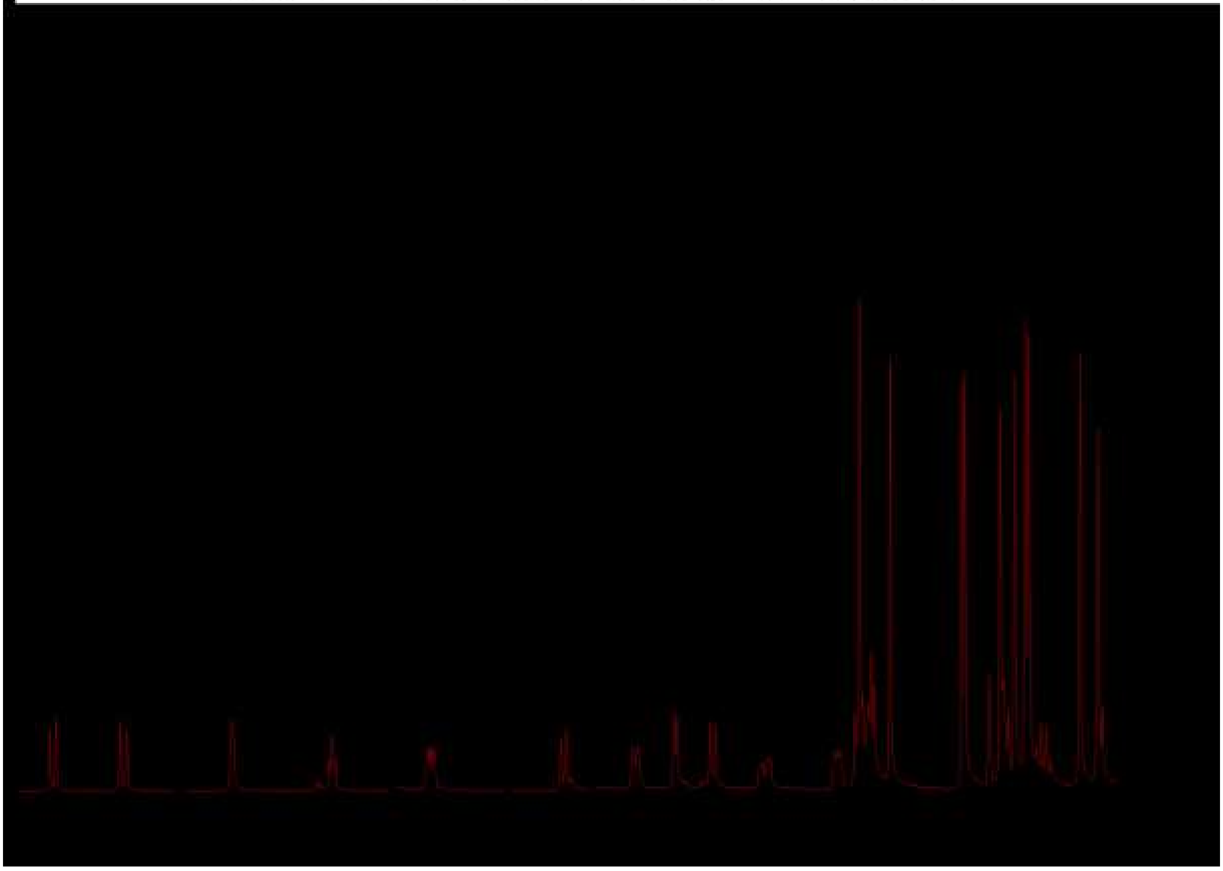

Supplementary Figure 2. HR-ESI/MS and ^{1}H-NMR results of compound 1 (16-*O*-acetyl cucurbitacin B). **a**, Negative HR ESI-MS *m/z* 645.3282 [M+ formic acid-H]$^-$ (calculated for $C_{35}H_{49}O_{11}$ 645.3275) and (**b**) ^{1}H-NMR (600 MHz, CD_3OD, δ_H) in Table 2.

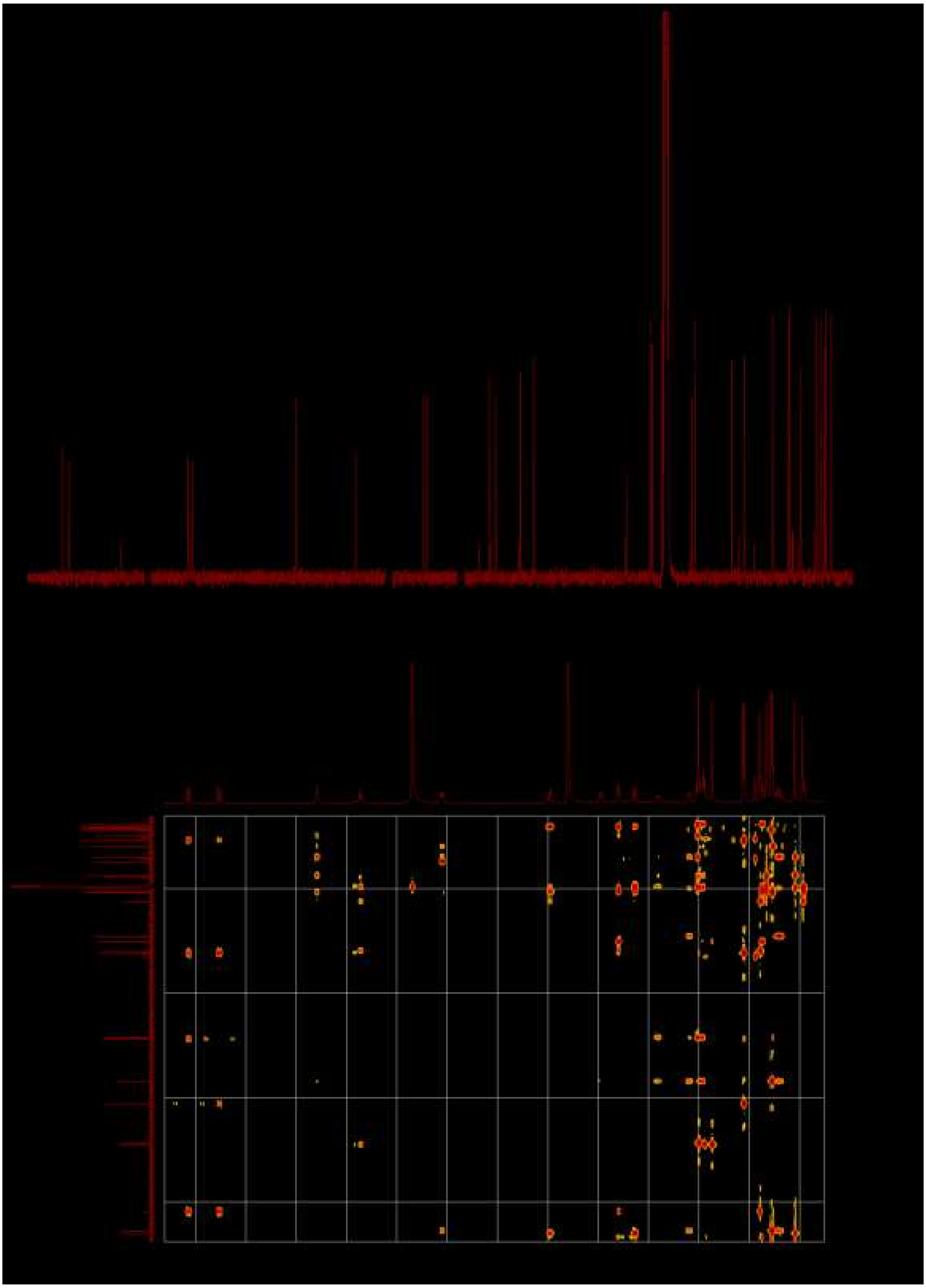

Supplementary Figure 3. ^{13}C-NMR and HMBC results of compound 1 (16-*O*-acetyl cucurbitacin B). a, ^{13}C-NMR (150 MHz, CD_3OD, δ_C) in Table 3 and (**b**) HMBC spectrum (CD_3OD, 600MHz).

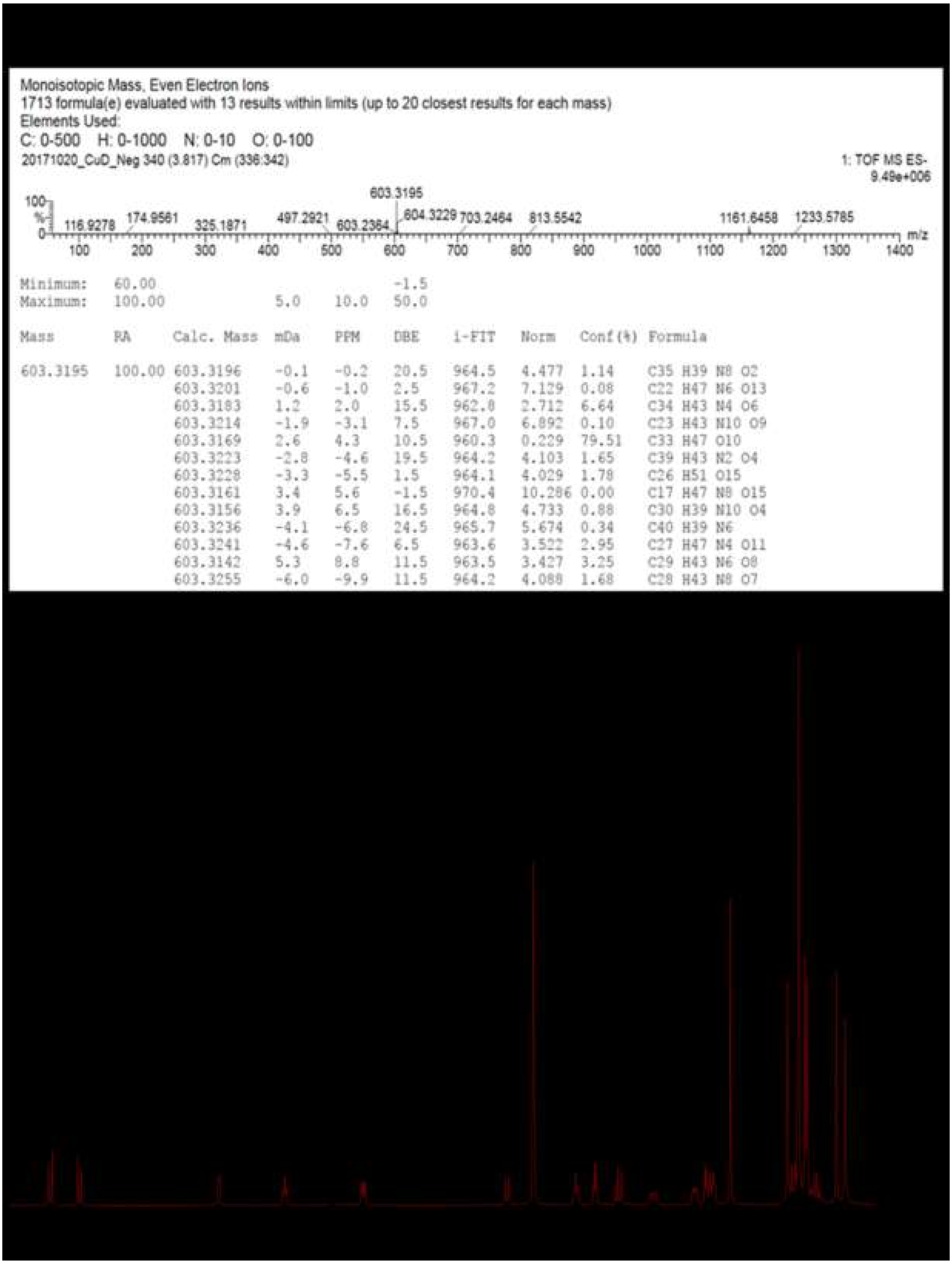

Minimum:	60.00				-1.5				
Maximum:	100.00		5.0	10.0	50.0				
Mass	RA	Calc. Mass	mDa	PPM	DBE	i-FIT	Norm	Conf(%)	Formula
603.3195	100.00	603.3196	-0.1	-0.2	20.5	964.5	4.477	1.14	C35 H39 N8 O2
		603.3201	-0.6	-1.0	2.5	967.2	7.129	0.08	C22 H47 N6 O13
		603.3183	1.2	2.0	15.5	962.8	2.712	6.64	C34 H43 N4 O6
		603.3214	-1.9	-3.1	7.5	967.0	6.892	0.10	C23 H43 N10 O9
		603.3169	2.6	4.3	10.5	960.3	0.229	79.51	C33 H47 O10
		603.3223	-2.8	-4.6	19.5	964.2	4.103	1.65	C39 H43 N2 O4
		603.3228	-3.3	-5.5	1.5	964.1	4.029	1.78	C26 H51 O15
		603.3161	3.4	5.6	-1.5	970.4	10.286	0.00	C17 H47 N8 O15
		603.3156	3.9	6.5	16.5	964.8	4.733	0.88	C30 H39 N10 O4
		603.3236	-4.1	-6.8	24.5	965.7	5.674	0.34	C40 H39 N6
		603.3241	-4.6	-7.6	6.5	963.6	3.522	2.95	C27 H47 N4 O11
		603.3142	5.3	8.8	11.5	963.5	3.427	3.25	C29 H43 N6 O8
		603.3255	-6.0	-9.9	11.5	964.2	4.088	1.68	C28 H43 N8 O7

Supplementary Figure 4. HR-ESI/MS and ^{1}H-NMR results of compound 2 (16-*O*-acetyl cucurbitacin D). **a**, Negative HR ESI-MS *m/z* 603.3195 [M+ formic acid-H]$^{-}$ (calculated for $C_{33}H_{47}O_{10}$ 603.3169) and (**b**) ^{1}H-NMR (600 MHz, CD_3OD, δ_H) in Table 2.

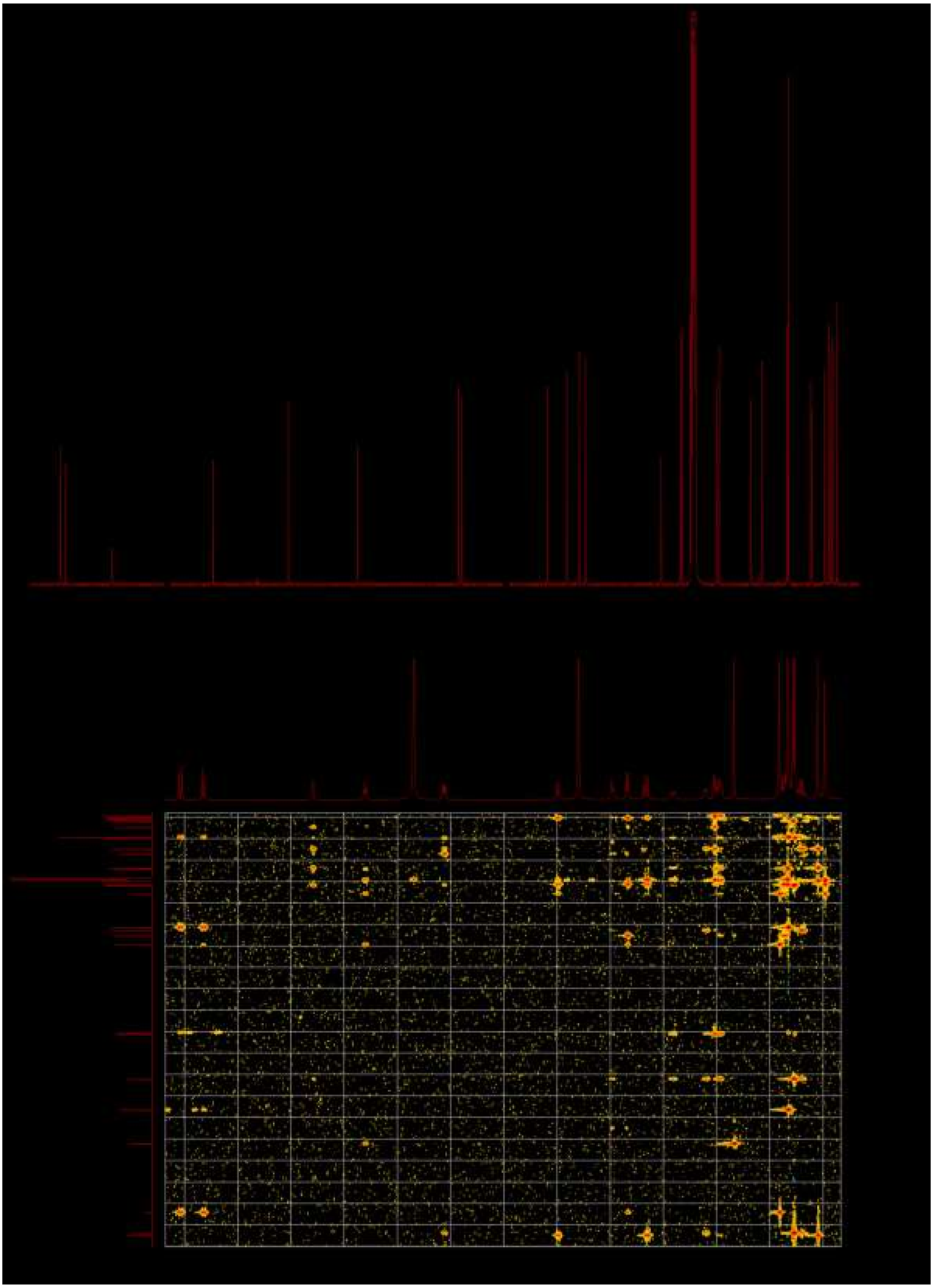

Supplementary Figure 5. ^{13}C-NMR and HMBC results of compound 2 (16-*O*-acetyl cucurbitacin D). **a**, ^{13}C-NMR (150 MHz, CD_3OD, δ_C) in Table 3 and (**b**) HMBC spectrum (CD_3OD, 600MHz).

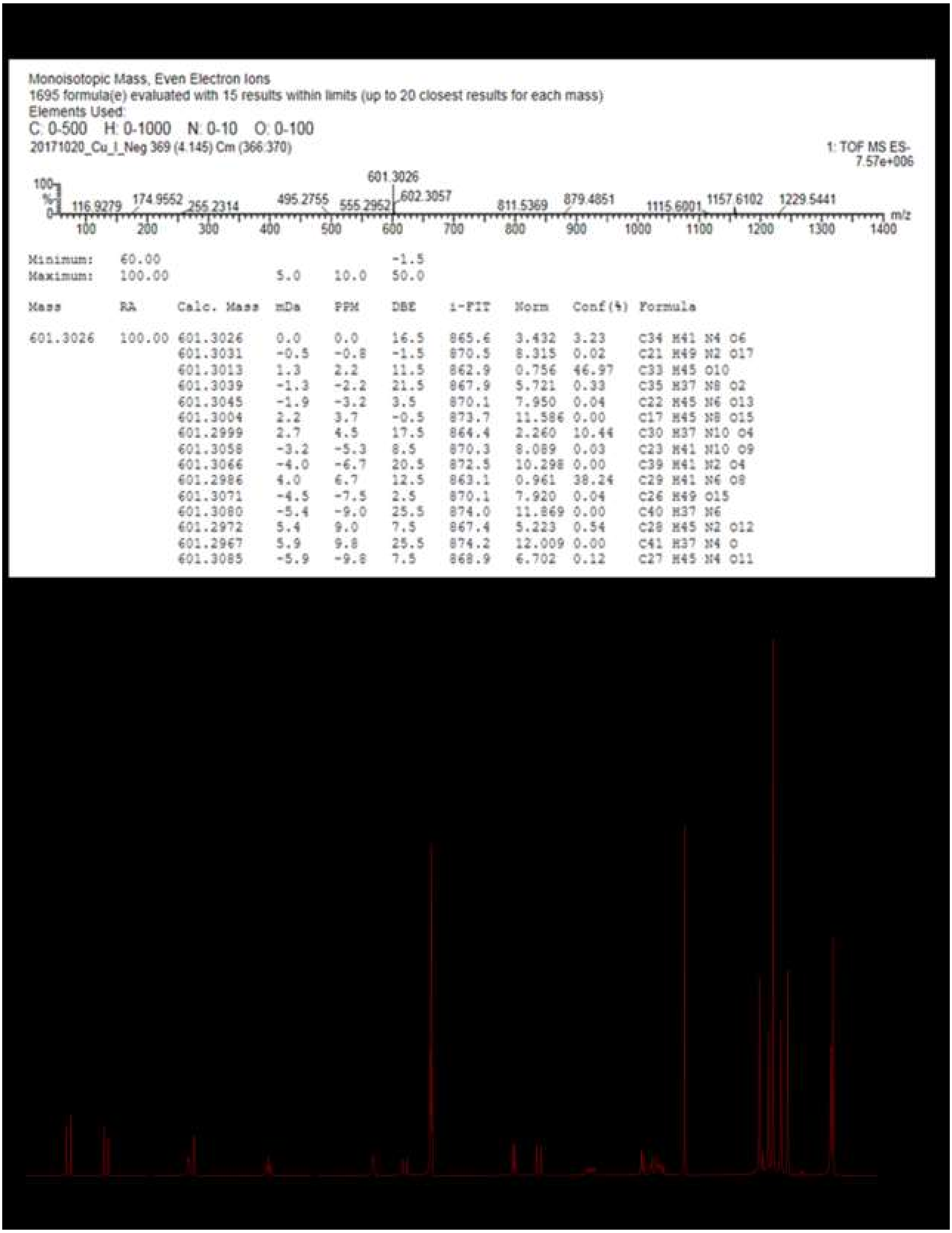

	Mass	RA	Calc. Mass	mDa	PPM	DBE	i-FIT	Norm	Conf(%)	Formula
Minimum:		60.00				-1.5				
Maximum:		100.00		5.0	10.0	50.0				
	601.3026	100.00	601.3026	0.0	0.0	16.5	865.6	3.432	3.23	C34 H41 N4 O6
			601.3031	-0.5	-0.8	-1.5	870.5	8.315	0.02	C21 H49 N2 O17
			601.3013	1.3	2.2	11.5	862.9	0.756	46.97	C33 H45 O10
			601.3039	-1.3	-2.2	21.5	867.9	5.721	0.33	C35 H37 N8 O2
			601.3045	-1.9	-3.2	3.5	870.1	7.950	0.04	C22 H45 N6 O13
			601.3004	2.2	3.7	-0.5	873.7	11.586	0.00	C17 H45 N8 O15
			601.2999	2.7	4.5	17.5	864.4	2.260	10.44	C30 H37 N10 O4
			601.3058	-3.2	-5.3	8.5	870.3	8.089	0.03	C23 H41 N10 O9
			601.3066	-4.0	-6.7	20.5	872.5	10.298	0.00	C39 H41 N2 O4
			601.2986	4.0	6.7	12.5	863.1	0.961	38.24	C29 H41 N6 O8
			601.3071	-4.5	-7.5	2.5	870.1	7.920	0.04	C26 H49 O15
			601.3080	-5.4	-9.0	25.5	874.0	11.869	0.00	C40 H37 N6
			601.2972	5.4	9.0	7.5	867.4	5.223	0.54	C28 H45 N2 O12
			601.2967	5.9	9.8	25.5	874.2	12.009	0.00	C41 H37 N4 O
			601.3085	-5.9	-9.8	7.5	868.9	6.702	0.12	C27 H45 N4 O11

Supplementary Figure 6. HR-ESI/MS and ^{1}H-NMR results of compound 3 (16-*O*-acetyl cucurbitacin I). **a**, Negative HR ESI-MS *m/z* 601.3026 [M+ formic acid-H]$^{-}$ (calculated for $C_{33}H_{45}O_{10}$ 601.3012) and (**b**) ^{1}H-NMR (600 MHz, CD_3OD, δ_H) in Table 2.

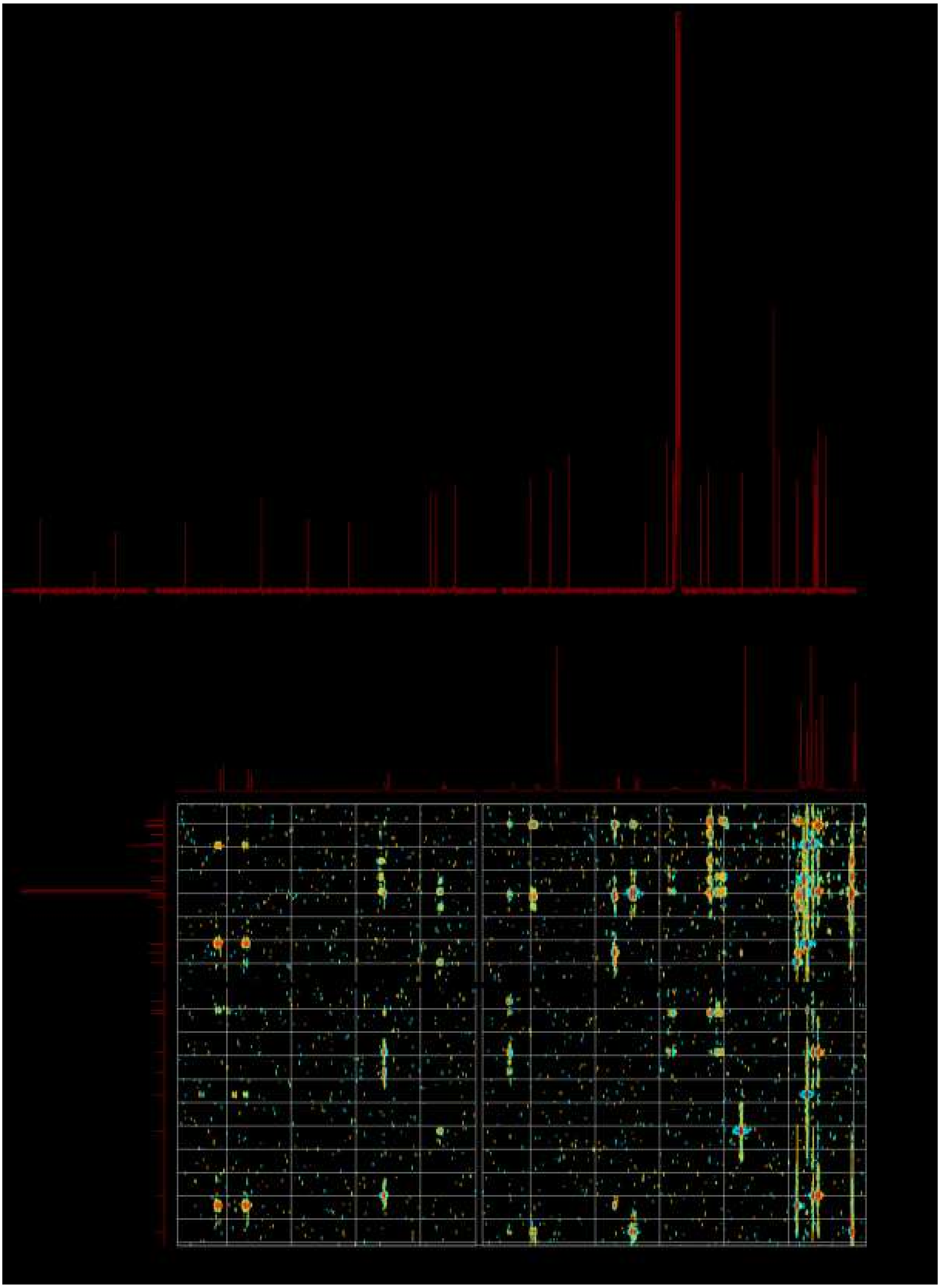

Supplementary Figure 7. ^{13}C-NMR and HMBC results of compound 3 (16-*O*-acetyl cucurbitacin I). **a**, ^{13}C-NMR (150 MHz, CD_3OD, δ_C) in Table 3 and (**b**) HMBC spectrum (CD_3OD, 600MHz).

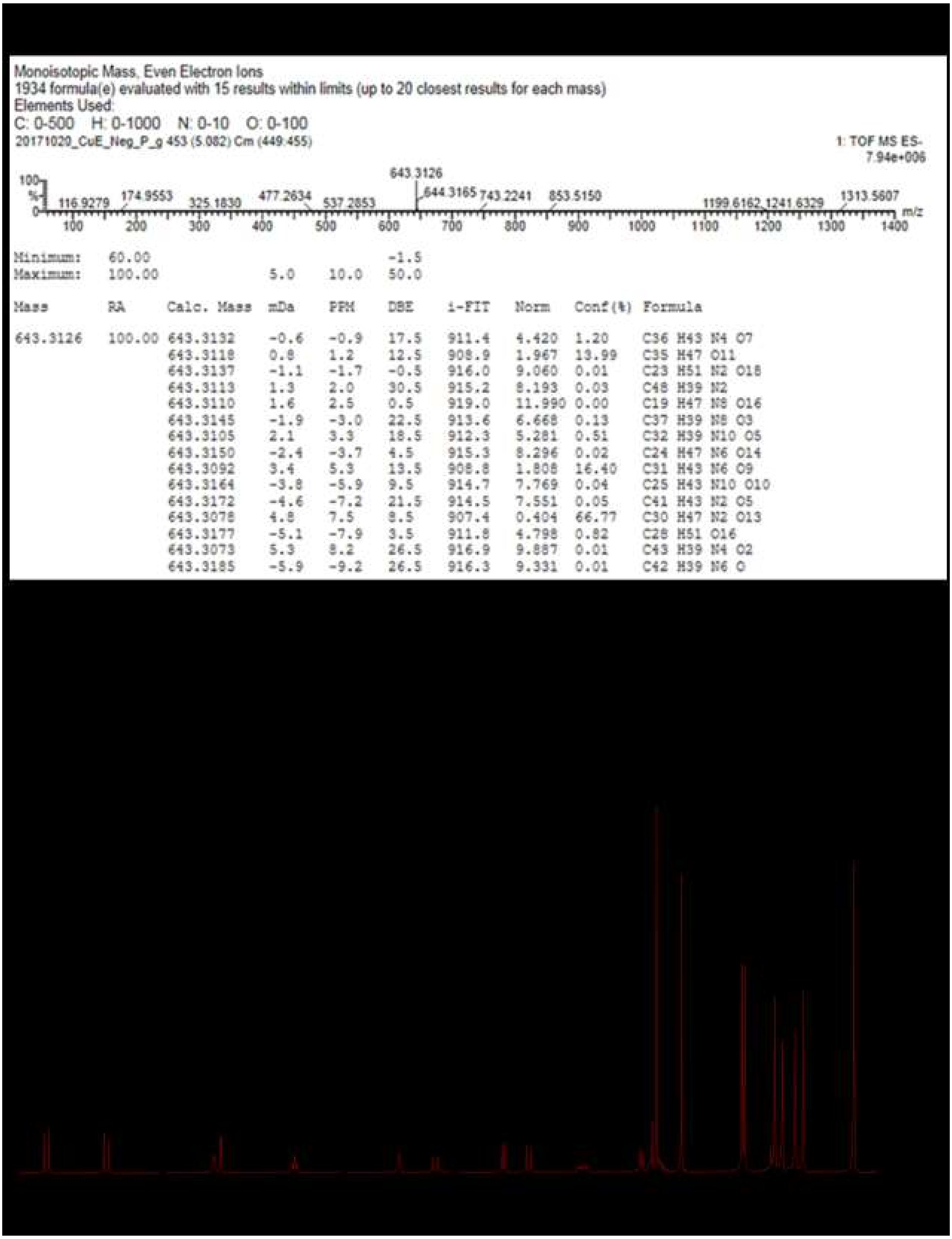

Minimum:	60.00				-1.5				
Maximum:	100.00		5.0	10.0	50.0				

Mass	RA	Calc. Mass	mDa	PPM	DBE	i-FIT	Norm	Conf(%)	Formula
643.3126	100.00	643.3132	-0.6	-0.9	17.5	911.4	4.420	1.20	C36 H43 N4 O7
		643.3118	0.8	1.2	12.5	908.9	1.967	13.99	C35 H47 O11
		643.3137	-1.1	-1.7	-0.5	916.0	9.060	0.01	C23 H51 N2 O18
		643.3113	1.3	2.0	30.5	915.2	8.193	0.03	C48 H39 N2
		643.3110	1.6	2.5	0.5	919.0	11.990	0.00	C19 H47 N8 O16
		643.3145	-1.9	-3.0	22.5	913.6	6.668	0.13	C37 H39 N8 O3
		643.3105	2.1	3.3	18.5	912.3	5.281	0.51	C32 H39 N10 O5
		643.3150	-2.4	-3.7	4.5	915.3	8.296	0.02	C24 H47 N6 O14
		643.3092	3.4	5.3	13.5	908.8	1.808	16.40	C31 H43 N6 O9
		643.3164	-3.8	-5.9	9.5	914.7	7.769	0.04	C25 H43 N10 O10
		643.3172	-4.6	-7.2	21.5	914.5	7.551	0.05	C41 H43 N2 O5
		643.3078	4.8	7.5	8.5	907.4	0.404	66.77	C30 H47 N2 O13
		643.3177	-5.1	-7.9	3.5	911.8	4.798	0.82	C28 H51 O16
		643.3073	5.3	8.2	26.5	916.9	9.887	0.01	C43 H39 N4 O2
		643.3185	-5.9	-9.2	26.5	916.3	9.331	0.01	C42 H39 N6 O

Supplementary Figure 8. HR-ESI/MS and ^{1}H-NMR results of compound 4 (16-*O*-acetyl cucurbitacin E). **a**, Negative HR ESI-MS *m/z* 643.3126 [M+ formic acid-H]$^{-}$ (calculated for $C_{35}H_{47}O_{11}$ 643.3118) and (**b**) ^{1}H-NMR (600 MHz, CD_3OD, δ_H) in Table 2.

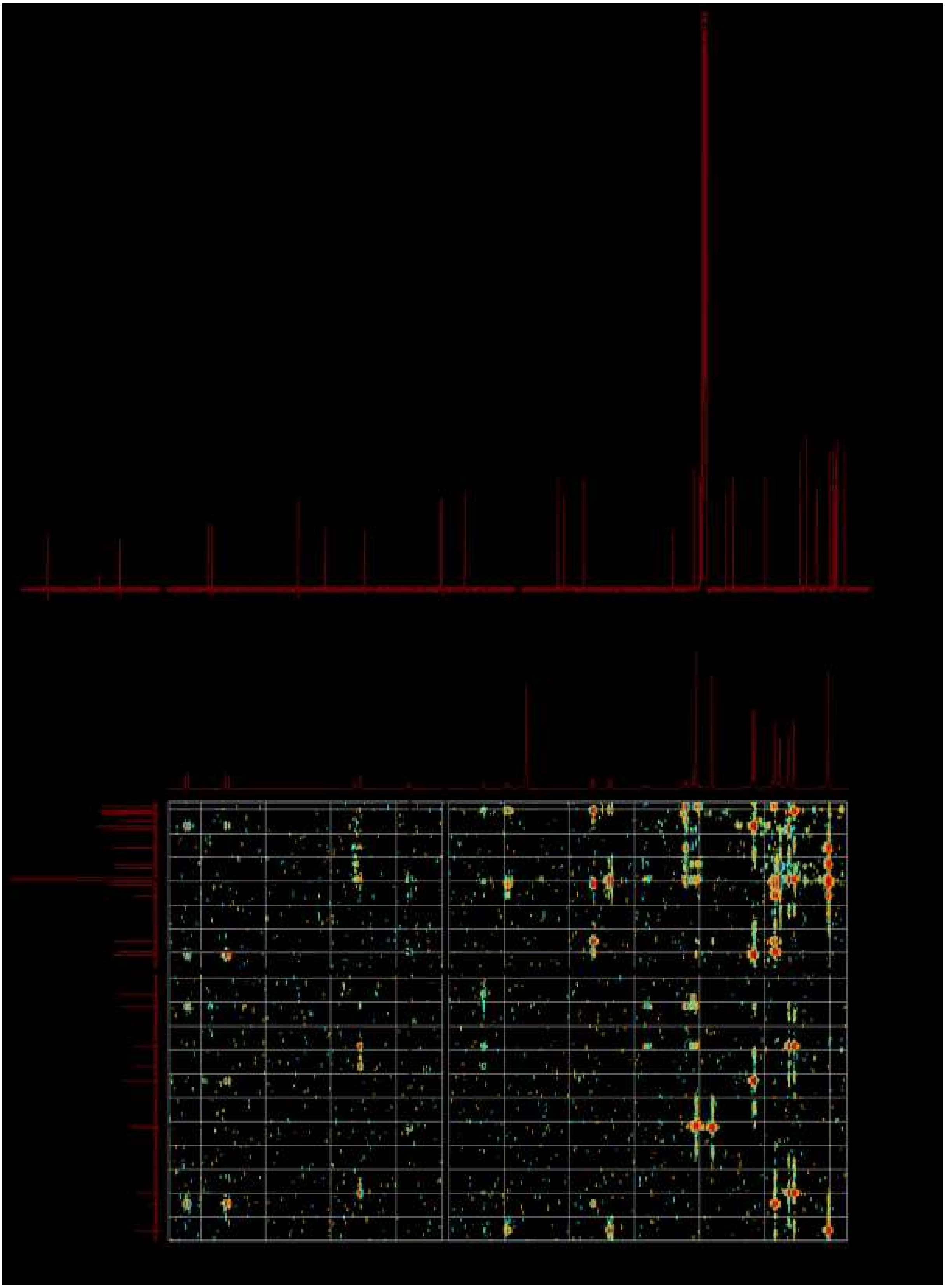

Supplementary Figure 9. ¹³C-NMR and HMBC results of compound 4 (16-*O*-acetyl cucurbitacin E). **a**, ¹³C-NMR (150 MHz, CD_3OD, δ_C) in Table 3 and (**b**) HMBC spectrum (CD_3OD, 600MHz).

Monoisotopic Mass, Even Electron Ions
2959 formula(e) evaluated with 25 results within limits (up to 20 closest results for each mass)
Elements Used:
C: 0-500 H: 0-1000 N: 0-10 O: 0-100
20171020_CuE_Glu_Neg 326 (3.663)   1: TOF MS ES- 1.49e+006

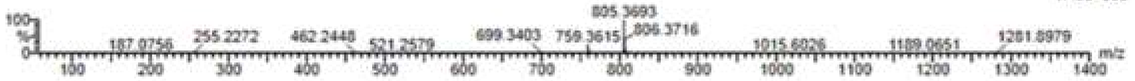

Mass	RA	Calc. Mass	mDa	PPM	DBE	i-FIT	Norm	Conf(%)	Formula
Minimum:	60.00				-1.5				
Maximum:	100.00		5.0	10.0	50.0				
805.3693	100.00	805.3692	0.1	0.1	10.5	508.5	7.016	0.09	C31 H53 N10 O15
		805.3700	-0.7	-0.9	22.5	512.8	11.250	0.00	C47 H53 N2 O10
		805.3682	1.1	1.4	35.5	516.4	14.918	0.00	C59 H49 O3
		805.3705	-1.2	-1.5	4.5	508.5	6.987	0.09	C34 H61 O21
		805.3678	1.5	1.9	5.5	510.6	9.079	0.01	C30 H57 N6 O19
		805.3673	2.0	2.5	23.5	510.9	9.400	0.01	C43 H49 N8 O8
		805.3714	-2.1	-2.6	27.5	513.4	11.879	0.00	C48 H49 N6 O6
		805.3719	-2.6	-3.2	9.5	505.4	3.856	2.12	C35 H57 N4 O17
		805.3665	2.8	3.5	0.5	512.3	10.831	0.00	C29 H61 N2 O23
		805.3660	3.3	4.1	18.5	510.3	8.818	0.01	C42 H53 N4 O12
		805.3727	-3.4	-4.2	32.5	514.3	12.809	0.00	C49 H45 N10 O2
		805.3655	3.8	4.7	36.5	515.8	14.259	0.00	C55 H45 N6 O
		805.3732	-3.9	-4.8	14.5	501.5	0.025	97.55	C36 H53 N8 O13
		805.3647	4.6	5.7	13.5	510.2	8.660	0.02	C41 H57 O16
		805.3740	-4.7	-5.8	26.5	516.4	14.856	0.00	C52 H53 O8
		805.3641	5.2	6.5	31.5	516.2	14.730	0.00	C54 H49 N2 O5
		805.3638	5.5	6.8	1.5	515.5	13.979	0.00	C25 H57 N8 O21
		805.3751	-5.8	-7.2	1.5	515.8	14.292	0.00	C24 H57 N10 O20
		805.3633	6.0	7.4	19.5	508.5	6.956	0.10	C38 H49 N10 O10
		805.3754	-6.1	-7.6	31.5	517.2	15.690	0.00	C53 H49 N4 O4

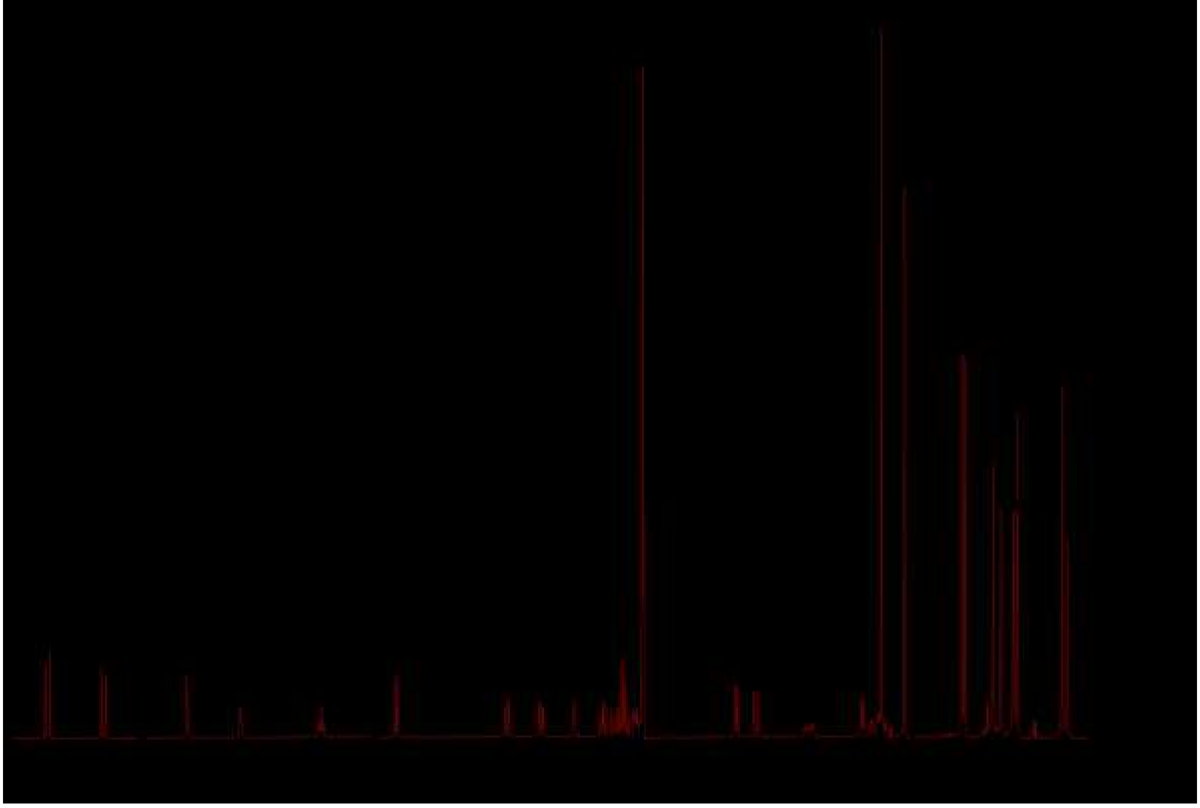

Supplementary Figure 10. HR-ESI/MS and ^{1}H-NMR results of compound 5 (2-*O*-*β*-D-glucopyranosyl 16-*O*-acetyl cucurbitacin E). **a**, Negative HR ESI-MS *m/z* 805.3693 [M+formic acid-H]$^{-}$ (calculated for $C_{41}H_{57}O_{16}$ 805.3646) and (**b**) ^{1}H-NMR (600 MHz, CD_3OD, δ_H) in Table 2.

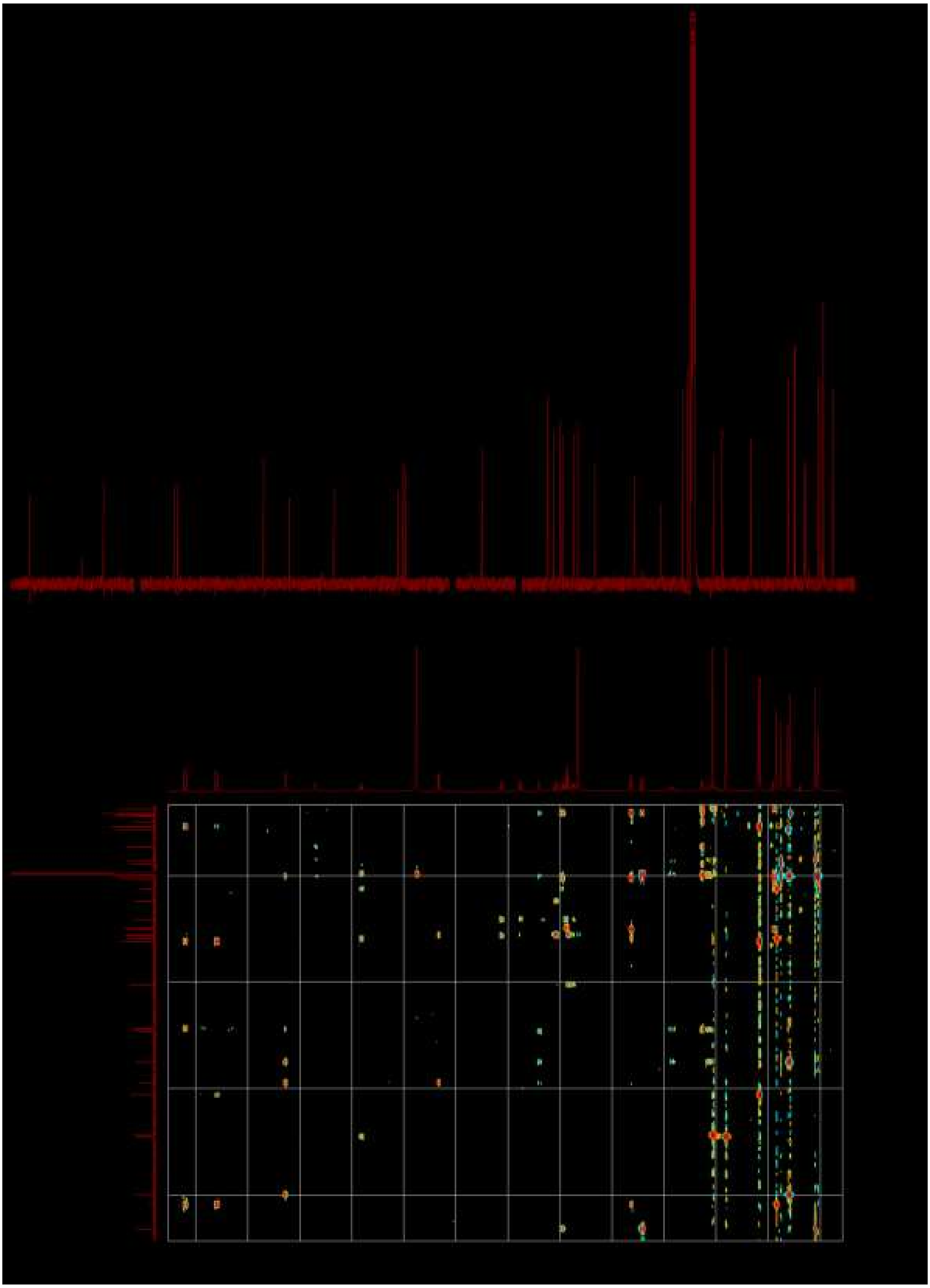

Supplementary Figure 11. ^{13}C-NMR and HMBC results of compound 5 (2-*O*-*β*-D-glucopyranosyl 16-*O*-acetyl cucurbitacin E). **a**, ^{13}C-NMR (150 MHz, CD_3OD, δ_C) in Table 3 and (**b**) HMBC spectrum (CD_3OD, 600MHz).

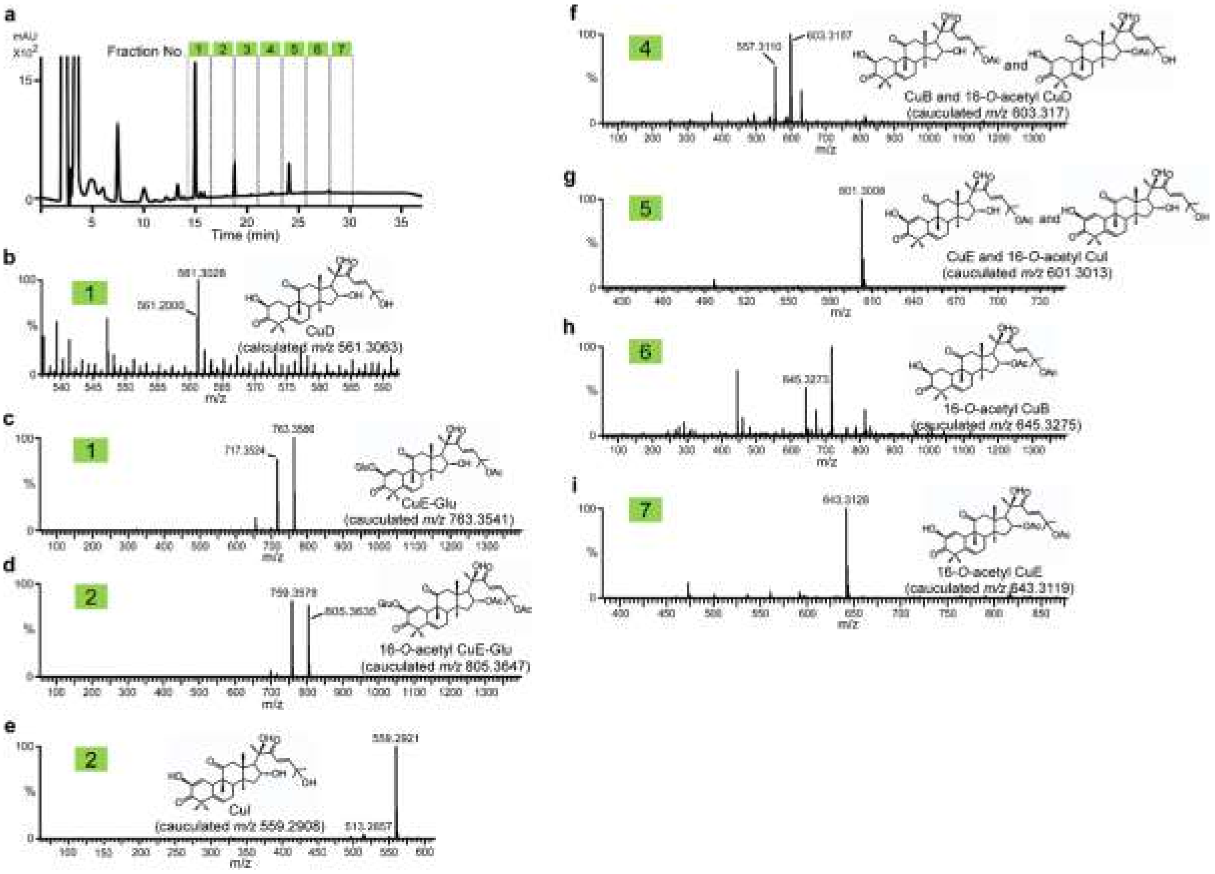

Supplementary Figure 12. Fractionation of watermelon extract during HPLC separation, and LC-MS analysis results of fractionated individual samples. a, HPLC chromatogram of watermelon extract for fractionations. **b**, LC–MS analysis of extract prepared from fraction number 1. The extracted ion chromatogram of the ion at *m/z* 561.3063 $[M+FA-H]^-$ corresponds to CuD. **c**, LC–MS analysis of extract prepared from fraction number 1. The extracted ion chromatogram of the ion at *m/z* 763.3541 $[M+FA-H]^-$ corresponds to CuE-Glu. **d**, LC–MS analysis of extract prepared from fraction number 2. The extracted ion chromatogram of the ion at *m/z* 805.3647 $[M+FA-H]^-$ corresponds to 16-*O*-acetyl CuE-Glu. **e**, LC–MS analysis of extract prepared from fraction number 2. The extracted ion chromatogram of the ion at *m/z* 559.2921 $[M+FA-H]^-$ corresponds to 16-*O*-acetyl CuI. **f**, LC–MS analysis of extract prepared from fraction number 4. The extracted ion chromatogram of the ion at *m/z* 603.3170 $[M+FA-H]^-$ corresponds to CuB and 16-*O*-acetyl CuD. **g**, LC–MS analysis of extract prepared from fraction number 5. The extracted ion chromatogram of the ion at *m/z* 601.3013 $[M+FA-H]^-$

corresponds to CuE and 16-*O*-acetyl CuI. **h**, LC–MS analysis of extract prepared from fraction number 6. The extracted ion chromatogram of the ion at *m/z* 645.3275 $[M+FA-H]^-$ corresponds to 16-*O*-acetyl CuB. **i**, LC–MS analysis of extract prepared from fraction number 7. The extracted ion chromatogram of the ion at *m/z* 643.3119 $[M+FA-H]^-$ corresponds to 16-*O*-acetyl CuE. Round brackets indicate calculated *m/z*.

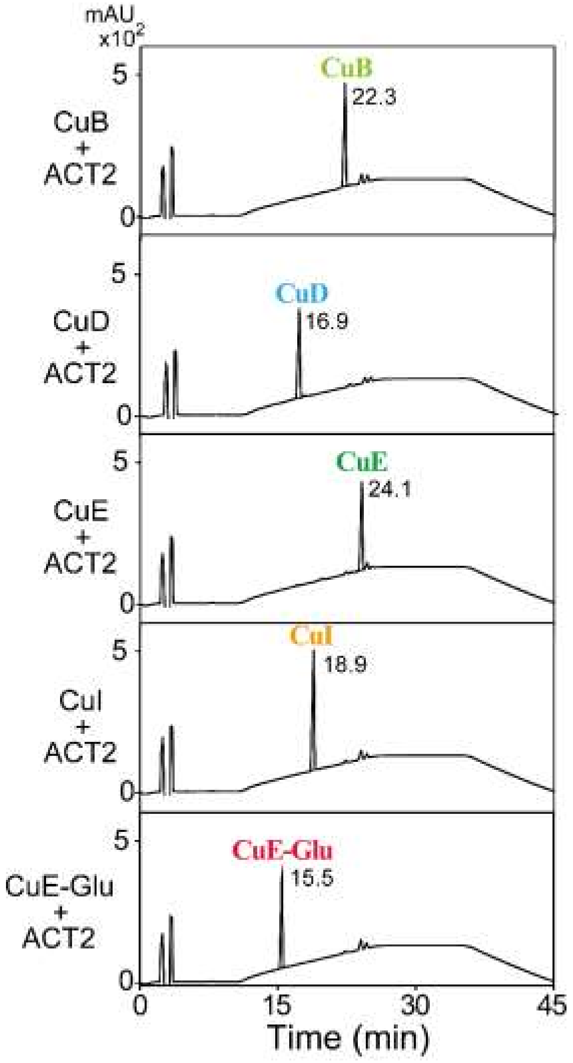

Supplementary Figure 13. ACT2 catalytic activity. HPLC analysis of ACT2 *in vitro* enzymatic reaction in the presence of the CuB, CuD, CuE, CuI and CuE-Glu which were used as a substrate.

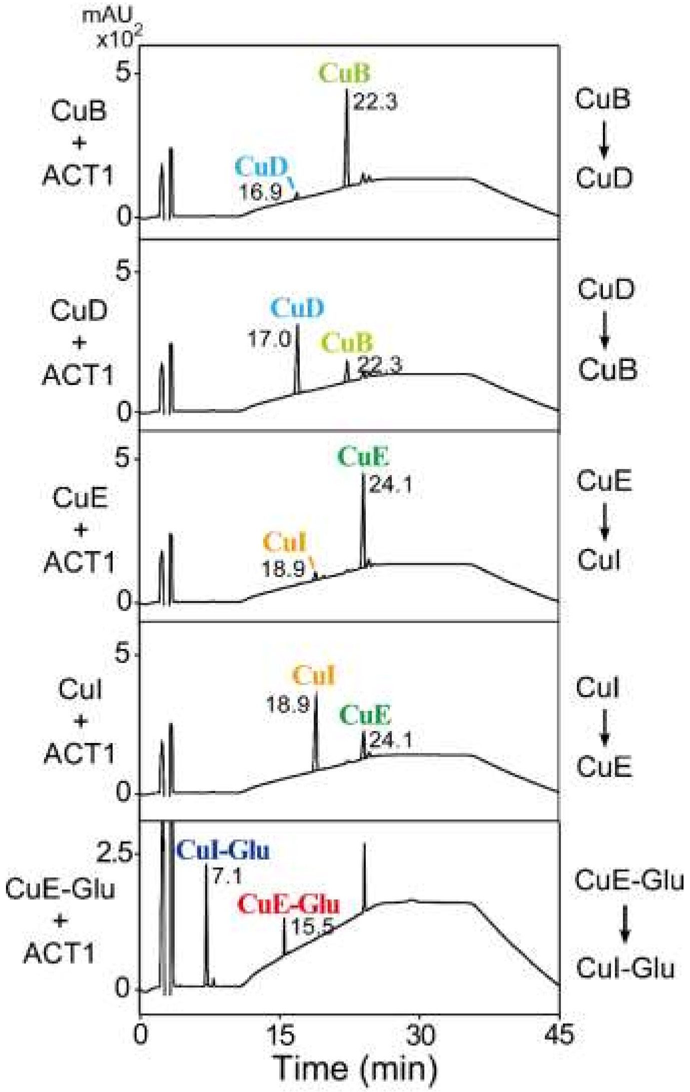

Supplementary Figure 14. ACT1 catalytic activity. HPLC analysis of ACT1 *in vitro* enzymatic reaction in the presence of the CuB, CuD, CuE, CuI and CuE-Glu which were used as a substrate.

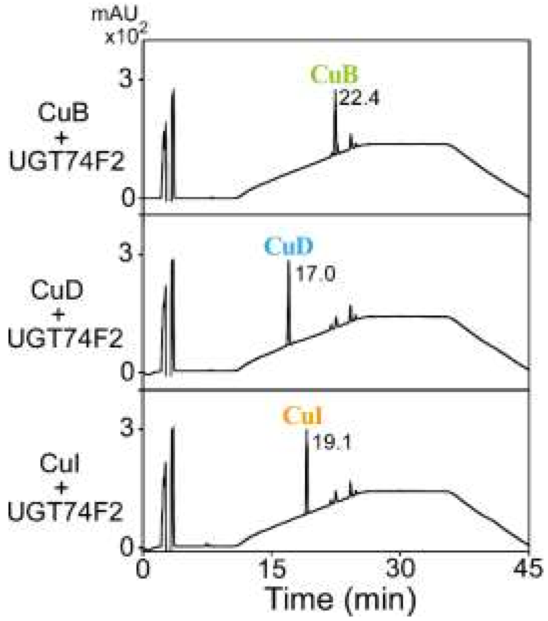

Supplementary Figure 15. UGT74F2 catalytic activity. HPLC analysis of UGT74F2 *in vitro* enzymatic reaction in the presence of the CuB, CuD and CuI which were used as a substrate.

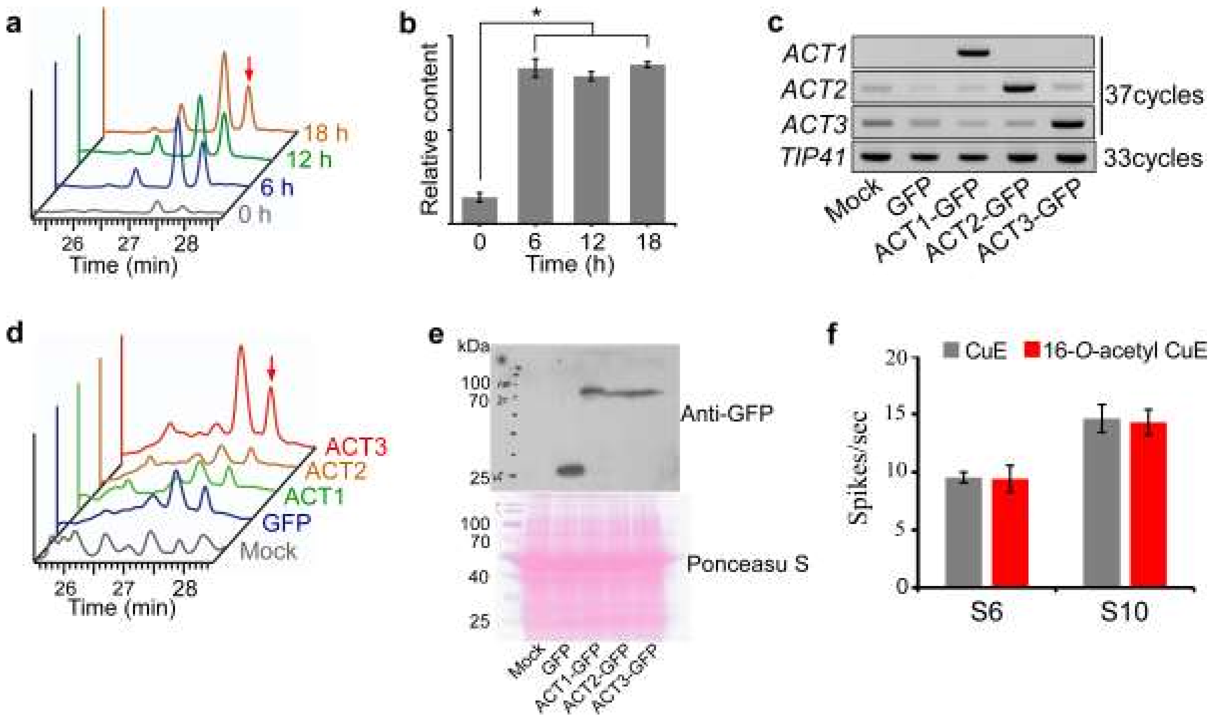

Supplementary Figure 16. Cucurbitacins accumulated by injury and *ACT3* transiently over expression, and evaluation of neuronal activation against *Drosophila*. a, HPLC chromatogram of 16-*O*-acetyl CuE in wounded leaves. **b**, Accumulation pattern of 16-*O*-acetyl CuE in wounded leaves were estimated by HPLC analysis. **c**, Semi RT-qPCR analysis results of leaves transiently overexpressed *ACTs* genes. **d**, HPLC chromatogram of 16-*O*-acetyl CuE in leaves transiently over expressed *ACT3* gene. **e**, Full immunoblot image of transiently overexpression *ACT* genes. **f**, The neuronal activation with CuE and 16-*O*-acetyl CuE. Average frequencies of action potential elicited from S6 and S10 sensilla (n=18-22). Red arrows indicate peaks of 16-*O*-acetyl CuE. The error bars represent ± SD (n = 3). Asterisks indicate significant difference (*, $P < 0.05$)

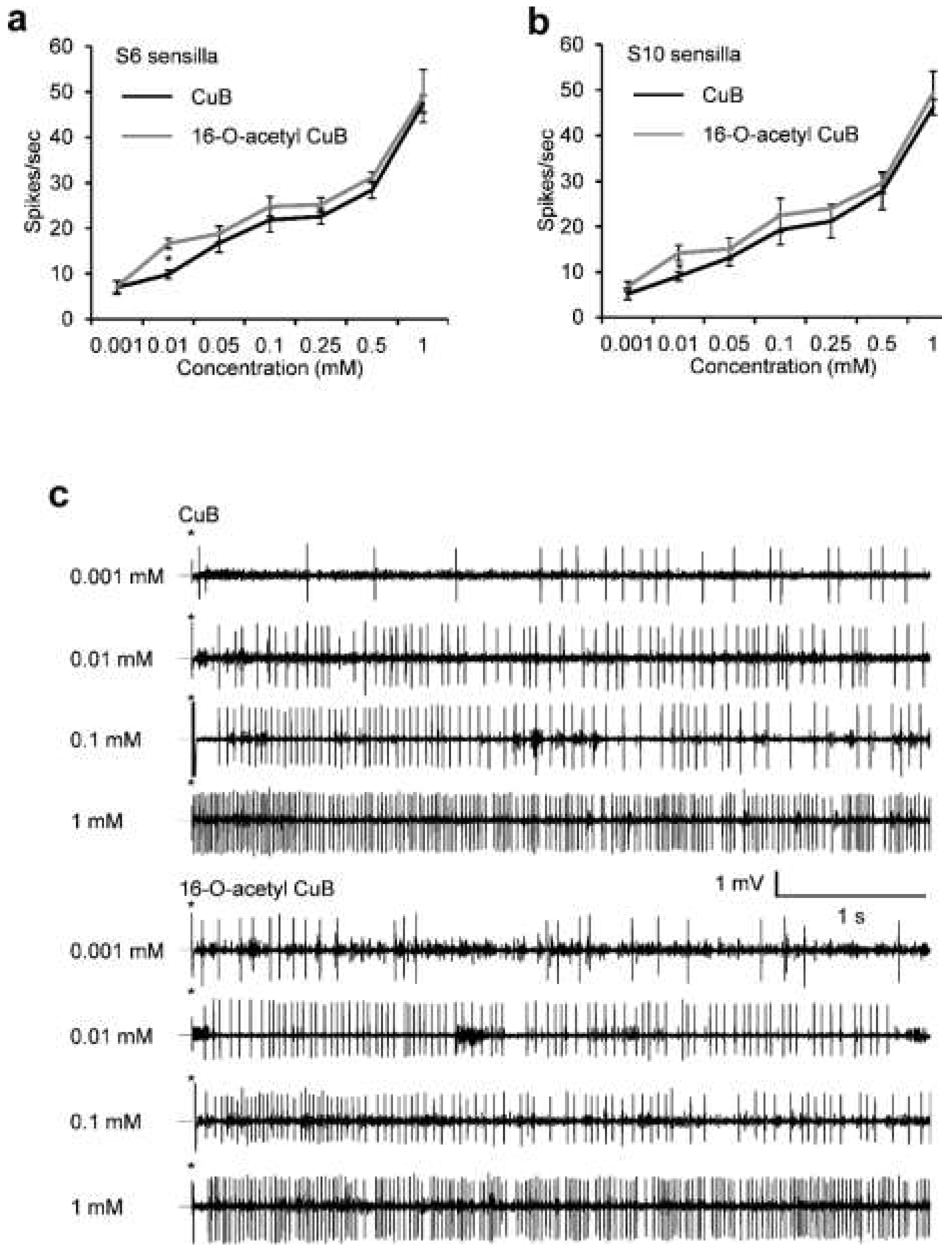

Supplementary Figure 17. The dose-dependent neuronal with CuB and 16-*O*-acety CuB. **a**, Average frequency of action potentials induced by the indicated concentraions of CuB and 16-*O*-acetyl CuB on S6 sensilla (n = 16-22). **b**, Average frequency of action potentials induced by the indicated concentrations of CuB and 16-*O*-acetyl CuB on S10 sensilla (n = 16-22). **c**, Representative sample traces obtained from S6 in **a**. All error bars represent SEMs. Single factor AVONA with Scheffe's ananlysis was used as a post hoc test to compare two sets of data. Asterisks indicate statistical significance compared with each genotype (*, $P < 0.05$).

Supplementary Tables

Supplementary Table 1. Summary statistics of sequencing data collected from seedling transcriptomes.

USDA ID	Total reads	Filtered reads	Filtered reads (%)	Reads mapped	Reads mapped (%)
PI532627	54,301,266	53,379,720	93.0	47,619,535	85.63
PI536451	52,981,816	51,980,816	92.6	46,892,579	85.50

Total reads: sequences of obtained sequencing process. Filtered reads: low quality reads filtered according to the criteria. Read mapped: map reads to genome.

Supplemental Table 2. Descriptions and changes of the up-regulated genes in PI532627 compare to PI536451 seedling.

Gene ID	Gene name	Description	Log2FC
Cla000422	*UGT74E2*	UDP-glycosyltransferase 74E2	3.22
Cla004119	*GLYT3*	Probable glycosyltransferase	1.13
Cla004392	*UGT74F2*	UDP-glycosyltransferase 74F2	3.23
Cla006564	*UGT76F1*	UDP-glycosyltransferase 76F1	2.48
Cla007080	*CPQ*	Cucurbitadienol synthase	4.65
Cla007081	*BAHD1*[a]	BAHD acyltransferase	3.55
Cla007082	*CYP87A3*[b]	Cytochrome P450 87A3	3.83
Cla007608	*crtN*	Dehydrosqualene desaturase	3.15
Cla008353	*BAHD1*[a]	BAHD acyltransferase	3.14
Cla008354	*CYP87A3*[b]	Cytochrome P450 87A3	4.74
Cla010352	*UGT90A1*	UDP-glycosyltransferase 90A1	4.09
Cla016525	*UGT83A1*	UDP-glycosyltransferase 83A1	1.60
Cla017252	*CYP705A5*	Cytochrome P450 705A5	3.25
Cla022004	*UGT74E2*	UDP-glycosyltransferase 74E2	2.32
Cla022651	*SE*	Squalene monooxygenase	2.48
Cla022713	*ACT*	Vinorine synthase	Inf.

"Inf." represent infinity. Log2FC: log2 fold change. *a*: genes described as same *CYP87A3* gene name. *b*: genes described as same *BAHD1* gene name.

Supplementary Table 3. Primer sequences used in this study.

Gene	Purpose	Sequence (5' to 3')	Direction
Cla000422	RT-qPCR	ACAGCTGAGAAAGGGTTGGT	F
		GCACTCCCAAACTCAATGCT	R
Cla004119	RT-qPCR	TGCATCTGTCCAAAGGGCTA	F
		GCTTCCCATTCCAACACCTC	R
Cla006564	RT-qPCR	ATGGTGGGTGAACGAGGATT	F
		ATCGGAACCCCTTCGCATAT	R
Cla007080	RT-qPCR	CATCCAGGCCATAGGACCAA	F
		CCCTTTATGCCAAACCACCC	R
Cla007082	RT-qPCR	GCTCTCCGCGACATTGAAAA	F
		CCGATGGATTCTCTGCAAGC	R
Cla007608	RT-qPCR	GTGGAGGTTGTGGTTTTCGA	F
		TGAAGACCATGAATCCGAGGT	R
Cla008354	RT-qPCR	GCCAGGCACAACTTACAACA	F
		TCATTGCCCGTTCCTTTAGC	R
Cla010352	RT-qPCR	TACAGCGATTTGGACCCAGT	F
		CGATCAATCCGTAGCTGCTG	R
Cla016525	RT-qPCR	CGTGGGAAGATTGTGGGTTG	F
		GTACGGCCAACACAGGAATC	R
Cla017252	RT-qPCR	GCGGTAGTGAAGGAGTGTCT	F
		GATCCACTGCAACCATGGTG	R
Cla022004	RT-qPCR	AGGGTGAAGCTGGATGAACA	F
		CCACCTTCATCCATGGCTTC	R
Cla022651	RT-qPCR	GGCATCTGGATTGGAGCAAG	F
		CCGTTGCTGGGAAGAACATC	R
ACT1 (*Cla007081*)	RT-qPCR	GCTTCCAAAATCGCTTCCCT	F
		GAAATCCCGGACGTTGCTTT	R
	Semi RT-qPCR	GCTTCCAAAATCGCTTCCCT	F
		GAAATCCCGGACGTTGCTTT	R
	Recombinant protein	caaatgggtcgcggatccATGGAGTCAGCATTGAAA	F
		gtggtggtggtgctcgagGTGTTGGAGCTGAAGAAC	R
	Transient expression	aacacgggggactctagaATGGAGTCAGCATTGAAAG	F
		ttatatctccttggatccTGTGTTGGAGCTGAAGAACA	R
ACT2 (*Cla008353*)	RT-qPCR	TAGTAGTTGGAGCCGGTTCG	F
		GTGTCCGTTAGCACCACAAA	R
	Semi RT-qPCR	GCCAGGCACAACTTACAACA	F
		TCATTGCCCGTTCCTTTAGC	R

	Recombinant protein	caaatgggtcgcggatccATGGAAGTTCAAATTCTC	F
		gtggtggtggtgctcgagGGAAAGGACACTAGGGTT	R
	Transient expression	aacacgggggactctagaATGGAAGTTCAAATTCTCA	F
		ttatatctccttggatccTGGAAAGGACACTAGGGTTT	R
ACT3 (*Cla022713*)	RT-qPCR	GTTACTGTGGCGGCGTTTAA	F
		TGATTGTGTTGGAAGGCAGC	R
	Semi RT-qPCR	GTTACTGTGGCGGCGTTTAA	F
		TGATTGTGTTGGAAGGCAGC	R
	Recombinant protein	caaatgggtcgcggatccATGGGGACGATGAATTAC	F
		gtggtggtggtgctcgagATTGGCACTTGGGTTCAA	R
	Transient expression	aacacgggggactctagaATGGGGACGATGAATTACA	F
		ttatatctccttggatccTATTGGCACTTGGGTTCAAA	R
UGT74F2 (*Cla004392*)	RT-qPCR	AGTGAAGGTGGGTGAGGATG	F
		CTGCCACCTTTCCTAAGTGC	R
	Recombinant protein	caaatgggtcgcggatccATGGGTTTAGAAGGGAAA	F
		gtggtggtggtgctcgagAACACTTGGTATCTTGTC	R
TIP41 (*Cla016074*)	RT-qPCR	GCTCATGAGACTGAGGGACA	F
		CGAGAGCTTGAAACGTAGCC	R
	Semi-qPCR	GCCTTTGATGCTCTGACTGG	F
		CGAGAGCTTGAAACGTAGCC	R

Lower case indicate the plasmid DNA sequences.

Supplementary Methods

Plant material for RNA sequencing

Two *Citrullus lanatus* germplasms were used in this study. The seeds of PI532627 (USDA plant ID) and PI536451 (USDA plant ID) were kindly provided by the United States Department of Agriculture Germplasm Resources Information Network (USDA GRIN). Watermelon plants were grown in a growth chamber at 28°C under long-day conditions (photoperiod, 16 h : 8 h, light : dark) at a light intensity of 120 μmol m^{-2} s^{-1}. Seedlings were harvested 13 days after germination for RNA sequencing and RT-qPCR analysis.

Construction of RNA sequencing libraries

Libraries were prepared for 100 bp paired-end sequencing using the TruSeq RNA Sample Preparation Kit (Illumina). Specifically, mRNA was purified and fragmented from 2 μg of total RNA using oligo (dT) magnetic beads. The fragmented mRNAs were synthesized as single-stranded cDNAs through random hexamer priming. Double-stranded cDNA was prepared using single-stranded cDNA as a template for second-strand synthesis. After sequential end repair processes, A-tailing, and adapter ligation, cDNA libraries were amplified via polymerase chain reaction (PCR). The quality of these cDNA libraries was evaluated using the Agilent 2100 BioAnalyzer (Agilent) and quantified using the KAPA library quantification kit (Kapa Biosystems), according to the manufacturer's instructions.

RNA sequencing and transcriptome analysis

Paired-end sequencing (2 × 100 bp) was performed using Illumina HiSeq2500 (Illumina). Low-quality reads were filtered according to the following criteria; reads containing more than 10% skipped bases (marked as 'N's); reads containing more than 40% of bases with quality scores < 20; and reads where the average quality score for each read is < 20. The filtering process was

performed using in-house scripts. Filtered reads were mapped to the reference genome related to the species using the aligner[1]. Gene expression was measured with Cufflinks v2.1.1[2] using the gene annotation database for the species. Non-coding gene regions were excluded from the analysis of gene expression using the –mask option. To improve the accuracy of the measurement, multi-read-correction and frag-bias-correct options were applied. Default settings were used for all other options. Differential expression analysis was performed by Cuffdiff[3]. To enhance accuracy, multi-read-correction and frag-bias-correct options were applied. Default settings were used for all other options. DEGs were identified based on a q-value threshold less than 0.05 for correcting errors caused by multiple-testing[4]. The GO database classifies genes according to the three categories: Biological Process (BP), Cellular Component (CC), and Molecular Function (MF), and provides information on the function of genes. To characterize the genes identified from DEG analysis, a GO-based trend test was performed through the Fisher's exact test[5]. Selected genes with P-values < 0.001 following the test were regarded as statistically significant.

Elucidation of compound 1 (16-*O*-acetyl cucurbitacin B) structure by MS and NMR spectroscopy data analysis

The molecular formula of compound 1 was determined to be $C_{34}H_{48}O_9$ based on the negative ESI-MS *m/z* 645 $[M+formic acid-H]^-$ and high resolution ESI-MS *m/z* 645.3282 $[M+formic acid-H]^-$ (calculated for $C_{35}H_{49}O_{11}$ 645.3275) (Supplementary Fig. 2a). The ^{1}H-NMR spectrum (600 MHz, CD_3OD, δ_H) presented proton signals due to eight singlet methyls [δ_H 1.56 (H-27), δ_H 1.54 (H-26), δ_H 1.39 (H-30), δ_H 1.33 (H-29), δ_H 1.29 (H-21), δ_H 1.27 (H-28), δ_H 1.05 (H-19), and δ_H 0.96 (H-18)], two acetyl methyls [δ_H 2.01 and δ_H 1.88], three olefin methines [δ_H 5.79 (1H, br. d, J = 3.2 Hz, H-6); δ_H 7.06 (1H, d, J = 15.6 Hz, H-24); δ_H 6.76 (1H, d, J = 15.6 Hz, H-23)], including a double bond found to have a trans conformation from the coupling

constant (J = 15.6 Hz), and many methylenes and methines. The proton signals suggested that compound 1 **is** a pentacyclic triterpenoid with two acetyl groups (Supplementary Fig. 2b and Supplementary Table 3). The ^{13}C-NMR spectrum (150 MHz, CD_3OD, δ_C) presented 34 carbon signals including two acetyl groups (δ_C 171.8, 172.6, 22.0, 21.9). Also, the carbon signals of three ketones [δ_C 215.2 (C-3), δ_C 214.0 (C-11), and δ_C 204.6 (C-22)], one olefin quaternary [δ_C 142.2 (C-5)], three olefin methines [δ_C 153.0 (C-24), δ_C 121.9 (C-6), and δ_C 121.3 (C-23)], two oxygenated quaternaries [δ_C 81.0 (C-25) and δ_C 79.8 (C-20)], two oxygenated methines [δ_C 75.4 (C-2) and δ_C 73.0 (C-16)], four quaternaries [δ_C 52.0 (C-13), δ_C 51.7 (C-4), δ_C 49.9 (C-9), and δ_C 49.7 (C-14)], three methines [δ_C 56.3 (C-17), δ_C 43.9 (C-8), and δ_C 35.0 (C-10)], four methylenes [δ_C 49.9 (C-12), δ_C 44.5 (C-15), δ_C 37.2 (C-1), and δ_C 24.9 (C-7)], and eight methyls [δ_C 30.0 (C-28), δ_C 27.1 (C-27), δ_C 26.9 (C-26), δ_C 24.8 (C-21), δ_C 21.3 (C-29), δ_C 20.7 (C-19), δ_C 20.3 (C-18), and δ_C 19.4 (C-30)] were observed, indicating that compound 1 is a monoacetyl curcurbitacin B (Supplementary Fig. 3a and Supplementary Table 4). An oxygenated methine proton signal (H-16) was observed at 5.37 ppm due to downfield shifting, because of the esterification effect, which is usually observed around 4.30 ppm in cucurbitacin B[6]. This was confirmed from the cross peak between an oxygenated methine proton signal (δ_H 5.37, H-16) and an ester carbon signal of an acetyl group (δ_C 172.6) in the gHMBC spectrum (Supplementary Fig. 3b). By comparing the spectroscopic data with those in literature[7], compound 1 was confirmed to be a 16-*O*-acetyl cucurbitacin B, a facacein, which was previously isolated from *Echinocystis esiacea*[23].

Elucidation of compound 2 (16-*O*-acetyl cucurbitacin D) structure by MS and NMR spectroscopy data analysis

The molecular formula of compound 2 was determined to be $C_{32}H_{46}O_8$ based on the negative ESI-MS *m/z* 603 [M+formic acid-H]$^-$ and HR ESI-MS *m/z* 603.3195 [M+formic acid-H]$^-$

(calculated for $C_{33}H_{47}O_{10}$ 603.3169) (Supplementary Fig. 4a). The molecular weight of compound 2 (558 amu) was 42 amu less than compound 1 (600 amu), indicating that compound 2 had one acetyl group less than compound 1. The ^{1}H-NMR and ^{13}C-NMR spectra of compound 2 were very similar to those of compound 1, except for a shortage of signals arising from one acetyl moiety (Supplementary Figs. 4b and 5a). NMR spectroscopy data were similar to those of a cucurbitacin D, except for additional signals from one acetyl group (δ_H 1.83, 3H, s; δ_C 172.3, 21.2). Attachment of the acetyl group at C-16 was confirmed by a downfield shift of the oxygenated methine proton signal (H-16) to δ_H 5.30, which usually occurs at δ_H 4.3[6]. In the gHMBC spectrum (Supplementary Fig. 5b), an oxygenated methine proton signal δ_H 5.30 (H-16) showed cross-peaks with an ester carbon signal of an acetyl group (δ_C 172.3) and an oxygenated quaternary carbon signal δ_C 79.6 (C-20), indicating that the acetyl group was located at C-16. Taken together, compound 2 was identified as 16-*O*-acetyl cucurbitacin D, a novel compound.

Elucidation of compound 3 (16-*O*-acetyl cucurbitacin I) structure by MS and NMR spectroscopy data analysis

The molecular formula of compound 3 was determined to be $C_{32}H_{44}O_8$ based on the negative ESI-MS *m/z* 601 [M+formic acid-H]$^-$ and HR ESI-MS *m/z* 601.3026 [M+formic acid-H]$^-$ (calculated for $C_{33}H_{45}O_{10}$ 601.3012) (Supplementary Fig. 6a). The molecular weight of compound 3, 556 amu, was 2 amu less than compound 2 (558 amu) indicating that it possessed two hydrogens less than compound 2. The ^{1}H-NMR and ^{13}C-NMR spectra of compound 3 were very similar to those of compound 2, except for the loss of signal due to an oxygenated methine and methylene, and an additional signals owing to an oxygenated olefin quaternary (δ_C 147.0, C-2) and an olefin methine (δ_H 5.74, 1H, d, *J*=3.0 Hz, H-1; δ_C 116.7, C-1) (Supplementary Figs. 6b and 7a). The NMR data were similar to those of a cucurbitacin I, except for the additional

signal of one acetyl group (δ_H 1.84, 3H, s; δ_C 172.3, 20.9). Due to a downfield shift of an oxygenated methine proton (δ_H 5.30; H-16), the position of acetyl was found to be C-16 of aglycone. In the gHMBC spectrum (Supplementary Fig. 7b), an oxygenated methine proton signal δ_H 5.32 (H-16) showed cross-peaks with an ester carbon signal of an acetyl group (δ_C 172.3) and an oxygenated quaternary carbon signal δ_C 79.6 (C-20), a quaternary carbon signal δ_C 49.7 (C-14), and a methine carbon signal δ_C 56.0 (C-17), indicating that the acetyl group was located at C-16. Taken together, compound 3 was identified to be a 16-*O*-acetyl cucurbitacin I, which was a new compound.

Elucidation of compound 4 (16-*O*-acetyl cucurbitacin E) structure by MS and NMR spectroscopy data analysis

The molecular formula of compound 4 was determined to be $C_{34}H_{46}O_9$ based on the negative ESI-MS *m/z* 643 $[M+formic\ acid-H]^-$ and HR ESI-MS *m/z* 643.3118 $[M+formic\ acid-H]^-$ (calculated for $C_{35}H_{47}O_{11}$ 643.3118) (Supplementary Fig. 8a). The molecular weight of compound 4, 598 amu, was 42 more than compound 3 (556 amu), indicating that compound 4 has one more acetyl group than compound 3. The ^{1}H-NMR and ^{13}C-NMR spectra of compound 4 were very similar to those of compound 3, except for the addition of an acetyl group signal (δ_H 1.90, 3H, s; δ_C 172.5, 21.3) (Supplementary Figs. 8b and 9a). From the oxygenated methine proton of H-16, there was a downfield shift according to the esterification effect, and the position of the acetyl group was revealed to be C-16. The gHMBC spectrum (Supplementary Fig. 9b), with an oxygenated methine proton signal δ_H 5.40 (H-16), was found to correlate with an acetyl carbonyl carbon signal (δ_C 172.5), an oxygenated quaternary carbon signal δ_C 79.8 (C-20), and an quaternary carbon signal δ_C 51.5 (C-13). Taken together, compound 4 was identified as a 16-*O*-acetyl curcurbitacin E, which was previously isolated from *Bacopa monnieri*[24].

Elucidation of compound 5 (2-*O*-*β*-D-glucopyranosyl 16-*O*-acetyl cucurbitacin E) structure by MS and NMR spectroscopy data analysis

The molecular formula of compound 5 was determined to be $C_{40}H_{56}O_{14}$ based on the negative ESI-MS *m/z* 805 $[M+formic\ acid-H]^-$ and HR ESI-MS *m/z* 805.3693 $[M+formic\ acid-H]^-$ (calculated for $C_{41}H_{57}O_{16}$ 805.3646) (Supplementary Fig. 10a). The molecular weight of compound 5, 760 amu, was 162 more than compound 4 (598 amu), suggesting that compound 5 has one hexose moiety more than compound 4. The ^{1}H-NMR and ^{13}C-NMR spectra of compound 5 were very similar to those of compound 4, except for the additional hexose signals (Supplementary Figs. 10b and 11a). The sugar was determined to be a *β*-glucopyranose based on the chemical shift of the carbon signals due to an hemiacetal (δ_C 101.3, C-1'), four oxygenated methines (δ_C 75.3, C-2'; δ_C 77.7, C-3'; δ_C 70.7, C-4'; δ_C 78.3, C-5'), and an oxygenated methylene (δ_C 62.0, C-6') in the ^{13}C-NMR spectrum, as well the coupling constant of the anomer proton signal (J = 7.8 Hz). In the HMBC spectrum, the anomer proton signal (δ_H 4.66, H-1') presented a cross peak with the oxygenated olefin quaternary carbon signal (δ_C 147.4, C-2), indicating that the sugar was linked to the hydroxyl group at C-2 (Supplementary Fig. 11b). Therefore, compound 5 was identified as a 2-*O*-*β*-D-glucopyranosyl 16-*O*-acetyl cucurbitacin E, which was previously isolated from *Gratiola officinalis*[25].

Semi RT-qPCR

To examine the expression of *ACTs* and *UGT* in unwounded and wounded watermelon leaves, samples were collected at certain times after wounding treatment and stored until total RNA extraction. For semi RT-qPCR analysis, total RNA was extracted from unwounded and wounded watermelon leaves using a RiboEx Total RNA Kit (GeneAll). RNA quality was determined using a Nanodrop ND-2000 spectrophotometer (Nanodrop Technologies), and only

high-quality RNA samples (A260/A230>2.0 and A260/A280>1.8) were used for subsequent experiments. cDNA synthesis was performed with 5 μg of total RNA using a SuperiorScript III Master Mix in accordance with the manufacturer's instructions (Enzynomics). cDNA (2 μL) fragments were used as templates for semi RT-qPCR using gene-specific forward and reverse primers (Supplementary Table 3). Semi RT-qPCR analysis was performed using a T100 thermal cycler (Bio-Rad) using a PCR Master mix solution (*i*-Taq) (INtRON). One stably expressed *TIP41* (*Cla016074*) gene was used as a reference gene (Supplementary Table 3). All semi RT-qPCR experiments were performed in two biological replicates (independently harvested samples). PCR products were analyzed using agarose gel electrophoresis to determine the relative abundance of transcripts.

References

1 Trapnell, C., Pachter, L. & Salzberg, S. L. TopHat: discovering splice junctions with RNA-Seq. *Bioinformatics* **25**, 1105-1111 (2009).

2 Trapnell, C. et al. Transcript assembly and quantification by RNA-Seq reveals unannotated transcripts and isoform switching during cell differentiation. *Nat Biotechnol* **28**, 511-515 (2010).

3 Trapnell, C. et al. Differential gene and transcript expression analysis of RNA-seq experiments with TopHat and Cufflinks. *Nat Protoc* **7**, 562-578 (2012).

4 Benjamini, Y. & Hochberg, Y. Controlling the False Discovery Rate - a Practical and Powerful Approach to Multiple Testing. *J R Stat Soc B* **57**, 289-300 (1995).

5 Fisher, R. A. On the Interpretation of $\chi 2$ from Contingency Tables, and the Calculation of P. . *Journal of the Royal Statistical Society* **85**, 87-94 (1922).

6 Ayyad, S. E., Abdel-Lateff, A., Basaif, S. A. & Shier, T. Cucurbitacins-type triterpene with potent activity on mouse embryonic fibroblast from Cucumis prophetarum, cucurbitaceae. *Pharmacognosy Res* **3**, 189-193 (2011).

7 Ryu, J. S. & Lloyd, D. Cell Cytotoxicity of Sodium-Nitrite, Sodium-Nitroprusside and Roussins Black Salt against Trichomonas-Vaginalis. *Fems Microbiol Lett* **130**, 183-187 (1995).
